# Transcriptomic sequencing and expression verification of identified genes modulating the alkali stress tolerance and endogenous photosynthetic activities of industrial hemp plant

**DOI:** 10.1371/journal.pone.0326434

**Published:** 2025-06-25

**Authors:** Zeyu Jiang, Di Wang, Ye Che, Sikandar Amanullah, Ling Zhang, Siyuan Jie, Wei Yang, Mingze Wang, Lina Wang, Guochao Qi

**Affiliations:** 1 Daqing Branch of Heilongjiang Academy of Agricultural Sciences, Daqing, China; 2 Department of Horticultural Science, North Carolina State University, Mountain Horticultural Crops Research, and Extension Center, 455 Research Drive, Mills River, North Carolina, United States of America; Hainan University, CHINA

## Abstract

Hemp (*Cannabis sativa* L.) has a long cultivation history around the world. In northeast part of China, the alkaline soil geology severely reduces crop production. In this study, we tried to evaluate the impacts of alkali-induced stress on the photosynthetic status and physiological indices of hemp plants. The microscopic evaluation of endogenous ultrastructure clearly demonstrated significant oxidative damage to the structure of the photosynthetic tissues associated with the membrane, resulting from an increase in the levels of MGDG and DGDG. The deformed photosynthetic apparatus induced by alkali-stress significantly inhibited the biosynthesis process of photosynthetic pigments, causing 49.25%, 52.72%, 65.31%, and 28.13% loss in total Chl, Chl a, Chl b, and carotenoids, respectively. Meanwhile, the reduction in chlorophyll fluorescence parameters (Pn (74.62%), Gs (39.69%), and Tr (83.77%)) along with the obviously increased MDA (28.57%) and H_2_O_2_ (35.18%) content exhibited that the inhibitory effect of alkali-stress not only decreased the photosynthetic efficiency by intercepting the nutrient supply but also generated excessive ROS, resulting in oxidative stress. Transcriptomic analysis (RNA-sequencing) revealed the considerably enriched GO terms as well as KEGG pathways that exposed the significant DEGs. The qPCR expression evaluation of down-regulated chlorophyll biosynthesis-related major genes (*GOGAT* (*LOC115699366*) and *HEMA* (*LOC133032634*)) and photosystem-related major genes (*PSB* (*LOC115701338*) and *HCF* (*LOC115707994*)) exhibited important molecular evidence for modulating the photosynthesis activity of hemp plant under devastating mechanism of alkali-stress. However, the transcript patterns of photorespiration-related genes (*GOX* (*LOC115697365*) and *GDC* (*LOC115707082*)) showed a slower decreasing trend at late stress stage (at 24 ~ 48 h), and the transcription of *SGAT* gene (*LOC115699360*) was even enhanced by stress treatment at 48 h, probably in an attempt to adjust cellular carbon balance and elevate the antioxidant properties induced by alkali-stress.

## Introduction

Soil is a vital component of ecosystems, supporting plant life and acting as a reservoir for water and nutrients. The major issues of soil erosion, evapotranspiration, use of synthetic fertilizers, and heavy grazing produce unfavorable conditions that lead to the accumulation of excessive alkaline-salinity on the soil surface [1]. These conditions are becoming the most significant threat to the sustainable progress of agriculture and the ecological environment, as the biological yield and quality of crops are severely compromised by alkaline-sensitive mechanisms [2].

Photosynthesis is a system of biological processes by which plants convert typical sunlight energy into the chemical energy necessary for metabolism activity. The environmental fluctuations related stress often results in significant photoinhibition, which affects metabolic activities within lipid membranes and disrupts hormone degradation of plants [3]. This disruption can trigger significant metabolic syndrome, ion toxicity, and organelle dysfunction [4], which inhibit the photosynthesis rate and leaf expansion, ultimately resulting in wilting, yellowing, and stunted growth of the leaves [5]. Furthermore, the plasma membrane acts as a defensive obstacle between the cytoplasm and external environmental factors based on various membrane lipids to maintain the arrangement and its essential components, while modifications of the lipid composition occur regularly, especially the remodeling of mono-galactosediacylglycerol (MGDG) and di-galactosyldiacylglycerol (DGDG), which primarily form the membrane of green tissues [6]; however, when the DGDG levels upsurge under stress phase, the DGDG to MGDG ratio decreases to boost the proper stability of the cell membrane [7,8].

Alkalinity stress can be alleviated by adjusting the internal physiological functions linked to the morphological characters of plants, which are influenced by balanced carbohydrate and hormonal regulation [9]. The superoxide dismutase (SOD) and peroxidase (POD) levels regulate high activity of reactive oxygen species (ROS) and help protect the super-oxidized membrane structure by altering the enzymatic activities of antioxidants [10]. However, the plant health and degree of cells damaged by alkalinity stress can be evaluated by integrating balanced levels of pH, conductivity (REC), antioxidant activity, and malondialdehyde (MDA) contents [11]. Molecular regulation is far more intricate compared to physicochemical and biological activities [12], and the study of the regulatory transcriptional factors of various genes and encoding proteins to enhance tolerance or resistance against salt stress in crop plants by remodeling the cell-wall-linked enzymes and biosynthesis of hormonal genes has significantly increased [13,14].

Recently, genomics, transcriptomics, and proteomics studies have been performed to identify candidate functional genes that affect ion transport proteins, antioxidants, photosynthetic proteins, and transcription factors under alkalinity and salinity stresses [15–17]. However, genetic evaluation of the photorespiration modulatory pathways involved in stress conditions has caused big concern. Photorespiration has always been noticed as an inefficient process for consuming substantial ATP and reductant, the emission of CO₂ and NH₃ [18], and for photoprotective functions. Mainly, glycine and serine have been reported to promote senescence and glutathione synthesis, which acts as an antioxidant to prevent the cell injury induced by ROS activity under alkali stress [19,20]. Photorespiration could protect photosynthesis activity from photoinhibition through energy dissipation in C3 plants under stress [21]. Some essential enzymes in the photorespiration pathway show different roles in modulating the cell injury triggered by abiotic and biotic stresses [22,23]. E.g., ribulose-1, 5-bisphosphate carboxylase/oxygenase (Rubisco), which is responsible for catalyzing the transformation of inorganic carbon to biomass, may accumulate due to the salt stress [24,25]. Similarly, an enriched expression of photorespiratory peroxisomes enzyme serine:glyoxylate aminotransferases (AT1 and AT2) presented higher resilient towards downy mildew in the wild-type melon [26]. However, the expression of serine:glyoxylate aminotransferases (SGAT) was noticed to be suggestively triggered by induced salinity in sea daffodil (*Pancratium maritimum* L.) [27].

At present, the advent of next-generation sequencing has made it advantageous to utilize transcriptome analysis (RNA sequencing, RNA-seq) that enables the rapid measurement of messenger RNA (mRNA) expression [28]. RNA-seq is frequently used to explore the evolution process and expression variations of stress-responsive differentially expressed gene (DEG) in multiple crops, e.g., maize, rice, barley, and Arabidopsis [29,30]. An in-depth understanding of important biological and physiological processes linked to genetic regulatory pathways is critical for directing the proliferation of vegetative growth of crop plants, as evidenced by the research findings that DEGs are exclusively contributed to modulating the salt and alkali stress conditions at different developmental stages, antioxidant activities, transcriptional regulation, and nutrient transportation [31,32].

In China, Heilongjiang province is a typical alkalinized and salinized area that occupies the dominant production area for industrial hemp (*Cannabis sativa* L.) [33]. The soda-saline-alkali soils of this area are well known due to the dominant salt components (sodium bicarbonate and sodium carbonate). Up to the present time, very less molecular and genetic basis research has explained the roles of candidate genes and pathways modulating plants susceptibility and resistance to the salt stress, but specific information about the devastating mechanism of alkaline stress hemp crop is limited. Hence, in this study, we explored the impact of alkali-induced stress on the physiological indices and photosynthetic functions of hemp plants and functional candidate genes modulating the alkali-stress tolerance were dissected using the comparative transcriptomic analysis (RNA-sequencing).

## Materials and methods

### Experiment materials and treatments

The alkaline-susceptible variety “Qingma No.1 (QM1)” of hemp was taken as the experimental material. The seeds of the QM1 variety were granted by the Heilongjiang Academy of Agricultural Sciences (HAAS), Daqing Branch, and the research was performed in the hygienic laboratory of HAAS. Experiment was conducted in completely randomized design (CRD) using three repetitions of 250 mmol·L^−1^ of alkali (sodium bicarbonate (NaHCO_3_))-induced stress and control treatments (CK, without stress), respectively. In order to cultivate seedlings, varietal seeds were first placed in muslin bags, allowed to germinate for two days at 30 °C, and the photosynthetic and physiological indices were checked from the seedlings of comparative groups of alkali-stress and control treatment at different time intervals (6 h, 24 h, and 48 h).

The germinated seedlings that were all 20 cm tall were transferred to a hydroponic chamber by treating them with Hoagland solution having 250 mmol·L−1 of alkali stress for a total time period of 48 hours (h), and the solution without alkali stress was marked as the control treatment [34–35]. All the seedlings of comparative groups were allowed to grow at an optimal temperature of 22–25 °C with a light:dark cycle of 16:8 hours. Later, the stress-acclimatized and control group seedlings were replanted in solid substrate in pots, and actual morphological observations were observed from the growth status of plants at 6 h, 24 h, and 48 h intervals. Further, the cytological, metabolic, physiological, and photosynthetic activities were assessed from collected leaf samples, then transcriptomic data was examined, putative DEGs were sorted, and gene expression trends were analyzed for practical confirmation [5].

### Observation of ultrastructure of hemp leaves

The internal cytological characteristics of hemp seedling leaves were examined using transmission electron microscopy based on different magnification levels (3,000 × , 25,000 × , and 60,000×), as previously reported [5]. In short, the infected leaves were freshly sampled from alkali-induced and control treatments at various time durations, respectively. The sampled leaves were cut into 2 mm-wide pieces, and the prepared pieces were fixed with 1% osmium tetroxide and 2.5% solution (v/v), respectively. The tissues were embedded in EMBed-812 before being thin-sliced using Ultra-cut UCT (Leica) and examined under transmission electron microscope (H-7500, Hitachi, Tokyo, Japan) after being dried by using acetone solutions.

### Estimation of photosynthetic pigments and cell membrane permeability

The vital photosynthetic pigments (carotenoids, Chl, Chl a, and Chl b) were assessed using the earlier reported protocol of ethanol–acetone (80%) extraction [36]. In brief, a total of 0.3 gram of fresh leaf tissue samples were collected from different treatment of alkali stress and control groups, and dipped in ethanol–acetone solution (1:1) for 24 hours. The extract absorbance rate was measured by spectrophotometer (UV/VIS, DU520, Thermo Fisher Scientific, Delaware, DE, USA) and final readings were noted as mg·g−1 of fresh weight (FW) [37]. The lipids were extracted using the slightly modification method as earlier reported [38], and the composition of lipid content was calculated with the electrospray ionization technique using mass spectrometry (MS).

### Determination of photosynthetic fluorescence parameters

The chlorophyll fluorescence associated parameters (net photosynthetic rate (Pn), intercellular carbon dioxide (CO_2_) concentration (Ci), stomatal conductance (Gs), and transpiration rate (Tr)) were evaluated from collected hemp leaf samples of comparative groups of alkali stress and control treatments, using an automated hand-held photosynthesis system (LI-6400XT, LI-COR Biosciences, Lincoln, NE, USA), respectively [5].

### Determination of MDA, H_2_O_2_, and antioxidant contents

MDA contents were measured as nmol L−1 on a fresh weight (FW) basis by adopting the thiobarbituric acid method as earlier reported [39]. H_2_O_2_ content was deter-mined in g·kg−1 per FW based on absorption rate of 460 nm of the spectrophotometer device (UV/VIS, DU520, Thermo Fisher Scientific, Delaware, DE, USA) [40]. Glutathione was measured in mol·kg−1 per FW using the 5, 5-dithio-bis-(2-nitrobenzoic acid) method at absorption rate of 412 nm [41]. The endogenous peroxidase (POD) activity was similarly quantified through spectrophotometer device (UV/VIS, DU520, Thermo Fisher Scientific, Delaware, DE, USA) by following the minor modification in intensity of absorption rate at 240 nm intervals in the biochemical reaction [42].

### Determination of SGAT, Rubisco activity, and amino acid contents

The leaf protein was extracted from 100 mg of sampled leaf tissue using a mild heated extraction buffer (comprising of 25 mM of HEPES-KOH, 1 mM of MgCl_2_, 1 mM EDTA, 1 mM of KCl, 0.1 mM of phenylmethylsulfonyl fluoride, 10 mM of *β*-mercaptoethanol, and 10% of glycerol, with final pH of 7.60), and protein concentration was determined as earlier reported method [43]. SGAT activity was similarly measured by following the formerly reported method [44]. The derivatives were measured through high performance liquid chromatography (HPLC) with 2, 4-dinitrofluorobenzene (DNFB) as the pre-column derivatization agent, using ODS-C18 column, and detected by 360 nm wavelength, as reported by Shang et al. [45]. Endogenous Rubisco (ribulose-1,5-bisphosphate carboxylase/oxygenase, RuBP) activity was assessed by using the commercially available kit (Rubisco Kit) specifically designed to monitor the CO_2_ fixation through colorimetric change in the reaction mixture (Ge Ruisi, Suzhou, China) [46].

### Transcriptomic sequencing

Firstly, RNA was extracted from the sampled leaves of the hemp seedlings grown under different groups (CK, 6 h, 24 h, and 48 h), using TransZol Up Plus RNA purification Kit (TransGen Biotech Company, China). The extracted RNA integrity was evaluated by measuring its absorbance at specific wavelengths on a spectrophotometer (NanoDropTM, Thermo Fisher Scientific, Delaware, DE, USA). Then, mRNA was enriched from total RNA using poly-T oligo-attached magnetic beads. AMPure XP beads was utilized to purify the double-strand cDNA templated from the mRNA, and size-selected, adaptor-ligated cDNA fragments were prepared for PCR and cDNA libraries performance, according to the formerly reported method [47]. The final libraries were developed from the sampled leaves at different time intervals of control and alkali-stressed groups using three biological replicates. All sequencing libraries were pooled up on an Illumina next-generation sequencing (NGS) platform. Finally, the obtained raw RNA-seq reads were uploaded on the NCBI website, under BioProject (Accession: PRJNA1163756, including three respective biological repetitions). The information of mapping statistics of reads for transcriptome data can be seen in S1 Table.

### Detection of differentially expressed genes (DEGs)

The relative gene expression was calculated using StringTie software utilizing fragments per kilobase of transcripts per million mapped reads (FPKM) and transcription levels of DEGs were validated. The gene expression differences were analyzed by DESeq software, by following threshold level at qValue < 0.05 and FoldChange > 2. The associated DEGs of collected samples at various time points were clustered and the functional enrichment analysis of KEGG and GO was conducted for investigating the high-level organized functions and integration of DEGs using an integrative bioinformatics software (TBtools-II) and a statistical computing program (R, version 4.3.0) [48,49]. The DEGs enrichment in KEGG pathways was determined by means of the KOBAS. The information of identified hub DEGs of chlorophyll synthesis, photorespiratory metabolism, and photosynthesis can be seen in S2 Table.

### Gene expression validation

For measuring the accuracy of obtained RNA-seq data and hub genes, the primers of candidate genes were exported using a bioinformatics software (Primer Premier, version 6.10) and real time quantitative polymerase chain reactions (RT-qPCR) were conducted for verification of genes expressions through 2-delta delta CT (2-∆∆CT) technique [50], and *Actin* was used as the standard “houskeeping” gene. The PCR reactions were performed on an Applied Biosystems Fast Real-time PCR System (ABI, 7500) using the following reaction processes: 95 °C for 10 min, 40 cycles at 95 °C for 15 s, 60 °C for 15 s, and 72 °C for 15 s, correspondingly. The information of exported primers of associated candidate DEGs is shown in S3 Table.

### Data analysis

We regularly recorded the photosynthetic and physiological parameter data from the cultivated seedlings under alkali-stress and control treatments. The suitable statistical analysis was done by using the software of the IBM-SPSS platform (version 28.0), and differences among the acquired results were perceived at significant statistical levels of p < 0.05 and p < 0.01 using Duncan’s test, as earlier reported statistical methods [5].

## Results

### Analysis of phenotypic characterization

**Fig 1:**
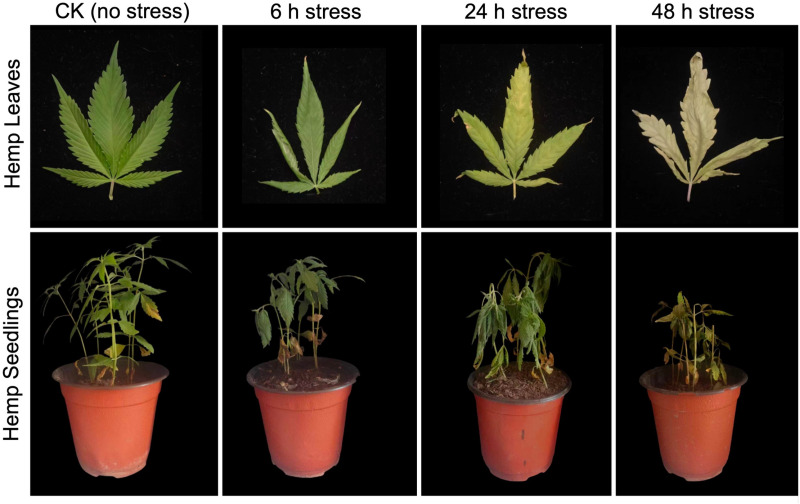
A pictorial view of hemp seedling and leaves grown under normal condition (CK, control without stress) and alkali-stress (NaHCO_3_ (250 mmol·L^−1^)) during three different time intervals (h, hours), respectively.

The visualized phenotypic characterization (Fig 1) depicted that the alkali stress had a crucial impact on developed stems and leaves of hemp; however, the leaves seemed as highly susceptible as compared to the stem tissue. Just as the results showed, the stem of hemp seedlings could keep straight under low alkali-induced conditions, while an obvious browning symptoms appeared in the stem under the extreme stress. In contrast, the significant inhibitory reaction in hemp leaves is supposed to be the main aspect reflecting the devastating mechanism of alkali stress. At the initial stage, no obvious chlorosis was observed except the dehydration-induced curled leaves; however, the exacerbation velocity of alkali stress was so rapid that it infected the entire leaf within 48 h, causing visible necrosis and extensive yellowing symptoms.

### Analysis of histological observations

**Fig 2:**
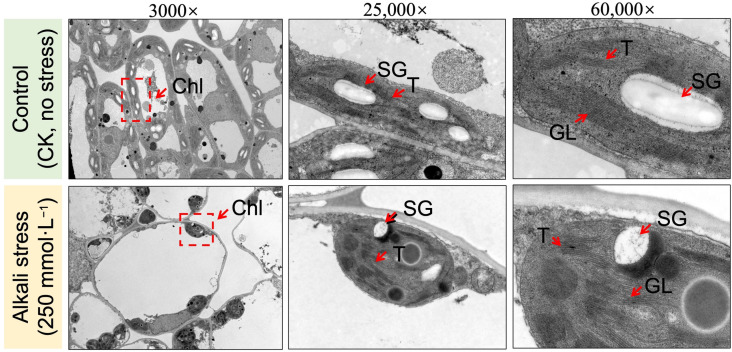
Microscopic observation of photosynthesis related endogenous ultrastructure in leaves of hemp seedling grown under control (CK, control without stress) and alkali stress (NaHCO₃ (250 mmol·L^−1^)) treatment at 48 h. Chl, chloroplast; T, thylakoid; GL, granal lamellae; SG, starch granule.

To assess the significant impact of alkali-induced stress on variation in the internal ultrastructure, we evaluated cytological characteristics by observing an endogenous structure of the leaf organelles of the full-grown seedling that was assessed under control and alkali stress based on transmission electron microscopy analysis (Fig 2). The seedlings grown under the control treatment group (without stress) depicted a normal arrangement of thylakoid chloroplast structure; however, an uneven thylakoid arrangement and swollen, oval-shaped structure of chloroplasts were observed along with decreased granal lamellae under the alkali-stressed treatment at 48 h. Additionally, the reduced starch granular quantity along with their integrity degradation indicated that the photosynthetic assimilation has been drastically damaged at 48 h of alkali-induced stress.

### Analysis of physiological and photosynthetic activities

**Fig 3:**
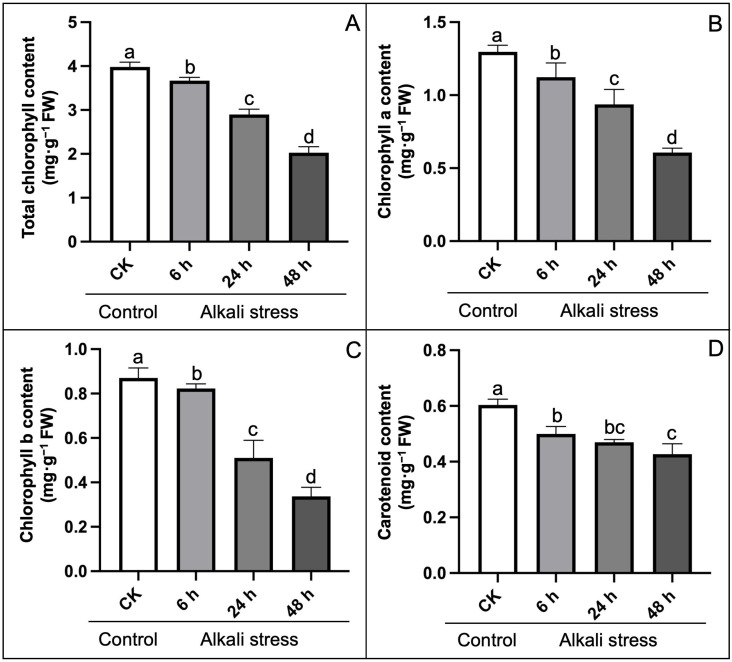
Effect of alkali-stress on photosynthetic pigments of hemp seedlings compared to control treatment. (A) Total chlorophyll content, (B) Chlorophyll a content, (C) Chlorophyll b content, (D) Carotenoid content. The statistical error bars indicate mean+SDs and dissimilar letters are denoting the significant change from each other (p < 0.05). Control (CK, without stress treatment).

We explored the physiological parameters associated with photosynthesis and membrane system (Fig 3). The results disclosed that the photosynthetic pigments gradually declined with the upsurged duration of alkali-stress infection effected by the internal chloroplast structure of leaves (Fig 2), which was in accordance with the upsurged distorted photosynthesis mechanism. Total chlorophyll (Chl), chlorophyll a (Chl a), chlorophyll b (Chl b), and carotenoid (Car) contents decreased by 49.25%, 52.72%, 65.31%, and 28.13%, respectively, at 48 h of time than that of the control (without stress), which may indicate that the dysfunction of photosynthesis apparatus induced by alkali stress severely inhibited the biosynthesis of photosynthetic pigments.

**Fig 4:**
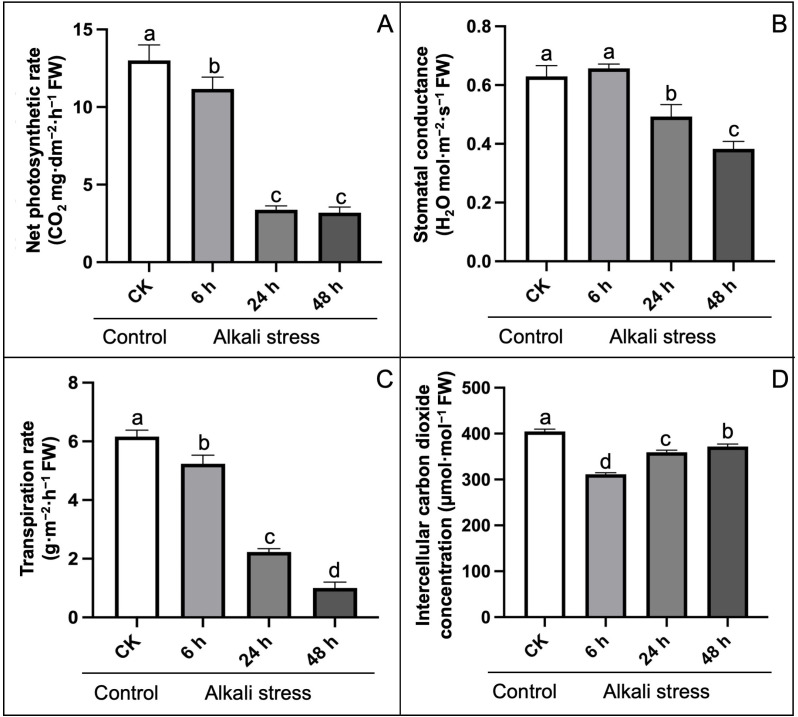
Effect of alkali-stress to chlorophyll fluorescence parameters compared to control treatment. (A) Net photosynthetic rate, (B) Stomatal conductance, (C) Transpiration rate, (D) Intercellular CO_2_ concentration. The statistical error bars indicate mean+SDs and dissimilar letters are denoting the significant change from each other (p < 0.05).

Then, we analyzed the changes in chlorophyll fluorescence related parameters of hemp leaves grown under alkali-induced stress compared to control treatment (Fig 4). The results exhibited that Pn, Gs, and Tr showed a stable decline trend under stress conditions, which decreased significantly by 74.62%, 39.69%, and 83.77% at 48 h com-pared with CK (Figs 4A–4C). In contrast, Ci showed a “V” type response with first decreasing and then increasing, while decreased by 8.39% compared with CK (Fig 4D).

**Fig 5:**
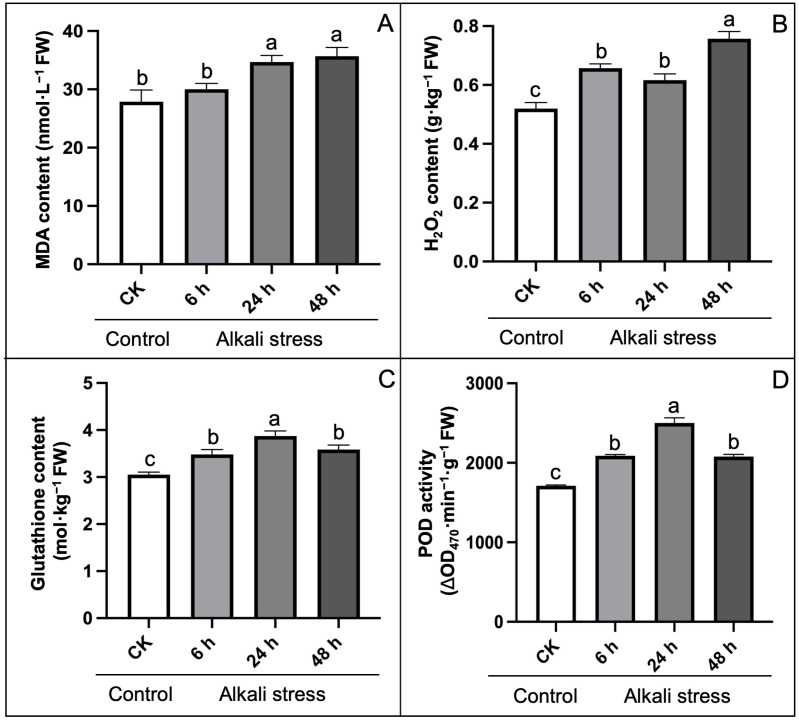
Effect of alkali-stress on physiological indices of antioxidant system compared to control treatment. (A) Malondialdehyde (MDA) content, (B) Hydrogen peroxide (H_2_O_2_) content, (C) Glutathione content, (D) Peroxidase (POD) activity. The statistical error bars indicate mean+SDs and dissimilar letters are denoting the significant change from each other (p < 0.05).

Further, the physiological indices related to lipid metabolism in structural membrane showed that MDA and H₂O₂ content were obviously higher in hemp leaves by 23.78% and 12.96% (at 24 h) and 28.57% and 35.18% (at 48 h) under alkali stress than CK groups (Figs 5A–5B), denoting the cell membrane damage. However, the response of H₂O₂ to alkali stress seems to be earlier than MDA, with a rapid increase of 16.67% at 6 h compared to control (without stress). This result was also supported by the fact that the amounts of MGDG and DGDG declined by 10.94% and 25.23%, respectively (Table 1), when compared to CK after 48 hours of alkaline stress. In addition, the MGDG/DGDG ratio showed an obvious upward trend with the deepening of alkali stress.

**Table 1:** Measurement of molar percentage (%) and ratio of MGDG and DGDG content of hemp seedling leaves grown under alkali-stress and control treatments at different time intervals, respectively.

Molar percentage (%)	Control (no stress)	Alkali stress (NaHCO_3_ (250 mmol·L^ − 1^))		
	CK	6 h	24 h	48 h
MGDG (%)	35.18 ± 0.94^a^	35.02 ± 0.31^a^	32.21 ± 0.61^b^	31.33 ± 1.27^b^
DGDG (%)	26.83 ± 0.40^a^	25.13 ± 0.31^a^	22.48 ± 0.60^b^	20.06 ± 1.13^b^
MGDG/DGDG	1.31 ± 0.03^c^	1.39 ± 0.03 ^b^	1.43 ± 0.06^b^	1.56 ± 0.15^a^

Due to an imbalance in the homeostasis of normal cellular and alkali-induced oxidative stress, glutathione content and POD activity upsurged significantly by 13.77% and 21.96% at 6 h compared to CK, but the antioxidant aptitude of hemp appeared to be inhibited with the duration of stress, e.g., 17.49% and 16.52% decline at 48 h compared to the maximum value at 24 h (Fig 5C–5D). These validations proposed that alkali stress negatively affected the growth and metabolism of hemp by inhibiting cell homeostasis. Meanwhile, the reduced stress-regulated capacity was consistent with the detected variations in cellular ultrastructure.

The substantial differentiations were observed for the SGAT and Rubisco activity as well as Gly and Ser content in hemp seedling leaves with the extension of alkali-stress interval (Table 2). The alkali-stress treatment caused a 7.95%, 15.75%, 69.69%, and 61.76% decrease in the amino acid activities and contents, respectively, at 48 h compared with CK. However, the alteration of these physiological indices exhibited a similar up-regulated trend at the initial stage, which may indicate that stress conditions activate the photorespiratory pathway.

**Table 2:** Effect of alkali-stress (NaHCO_3_ (250 mmol·L^−1^)) on SGAT activity, Rubico activity, glycine (Gly), and serine (Ser) content in hemp seedling leaves grown under different time intervals. Statistical letters are exhibiting the significant differences among the values.

Treatments		SGAT activity (mol·min^ − 1^·mg^ − 1^ FW)	Rubisco activity (nmol (CO_2_) min^ − 1^·mg^ − 1^ FW)	Gly content (μmol·g^ − 1^ FW)	Ser content (μmol·g^ − 1^ FW)
Control	CK	0.88 ± 0.03^a^	216.07 ± 4.45^b^	2.21 ± 0.05^a^	3.87 ± 0.12^a^
Alkali stress	6 h	0.93 ± 0.02^a^	232.45 ± 2.40^a^	2.14 ± 0.13^a^	4.16 ± 0.14^a^
	24 h	0.90 ± 0.03^a^	194.65 ± 2.11^c^	1.59 ± 0.02^b^	2.65 ± 0.06^b^
	48 h	0.81 ± 0.03^b^	182.14 ± 1.61^d^	0.67 ± 0.15^c^	1.48 ± 0.04^c^

### Analysis of transcriptomic (RNA-seq) data

We obtained good-quality sequencing reads for transcriptomic data analysis from collected samples of alkali-stressed groups and control (S1 Table): the T6 group (T6_1, T6_2, and T6_3), the T24 group (T24_1, T24_2, and T24_3), and the T48 group (T48_1, T48_2, and T48_3)”, and control (CK, without stress) treatment groups (CK_1, CK_2, and CK_3), respectively. The mapping statistics of RNA-seq reads of CK samples showed >90% of reads coverage (91.3% for CK_1, 91.1% for CK_2, 91.08% for CK_3). For the T6 treatment samples, the total mapped reads were 90.33% for T6_1, 89.05% for T6_2, and 91.65% for T6_3. For the T24 treatment samples, the total mapped reads were 62.57% for T24_1, 65.5% for T24_2, and 63.63% for T24_3. For the samples of T48 group treatment, the total mapped reads were 46.31% for T48_1, 90.76% for T48_2, and 40.06% for T48_3, individually.

### Analysis of detected DEGs

**Fig 6:**
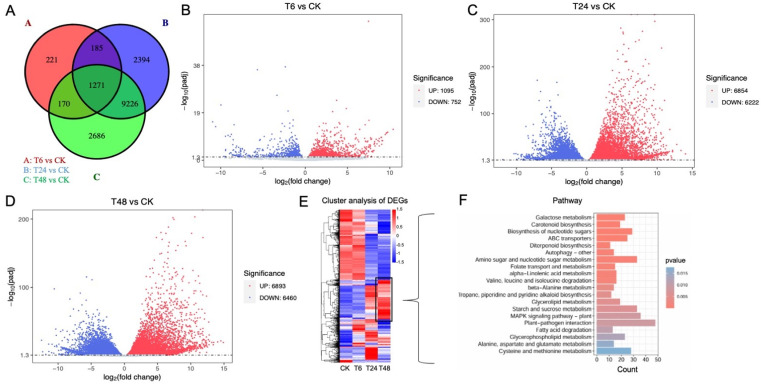
Analysis of categorized DEGs related to hemp leaves under comparative groups of alkali-stress and control treatments. (A) Venn diagram of DEGs in comparative treatment groups at different time intervals, (B) Volcano plot of DEGs between alkali-stress and control at 6 h, (C) at 24 h, (D) at 48 h, (E) Hierarchical clustering analysis of DEGs, (F) Pathway information of clustered gene involved in the T48 group of alkali-stress.

The Venn diagram (Fig 6A) exhibited a total of 1847, 13076, and 13353 genes that were expressed differentially in the comparative treatment groups of control and alkali-stress (T6_vs_CK, T24_vs_CK, and T48_vs_CK), which denoted the majority of DEGs were late-response genes. However, there were more up-regulated DEGs (such as 1095, 6854, and 6893) that were identified at different time intervals as compared with down-regulated DEGs (such as 752, 6222, and 6460) for hemp under alkali stress for 6 h, 24 h, and 48 h (Fig 6B–6D), and cluster analysis of DEGs similarly showed significant expressions (Fig 6E). As anticipated, the candidate DEGs identified in the T48 group (Fig 6F) exhibited a significant alteration trend at the transcriptomic level under alkali stress, that were enriched in, e.g., Galactose metabolism, Glycerolipid metabolism, and Starch and sucrose metabolism which are closely related to the membrane component and starch and sucrose metabolism.

### Analysis of GO terms and KEGG pathways

Gene ontology (GO) analysis results exhibited that the up-regulated DEGs (S1 Fig) at 6 h interval of time were enriched in transcription factors activity as well as photosynthetic pigments biosynthesis-related substrate and genes, while the down-regulated DEGs (S2 Fig) were considerably enriched in lipid metabolism as well as amino acid metabolism processes. Likewise, the up-regulated DEGs were enriched in hydrolase activity, transcription regulator activity, and organic substance metabolism; the down-regulated DEGs were enriched in amide metabolic process, membrane component, and photosynthesis at 24 h and 48 h stress.

Further, the detected DEGs were found to correspond to 130 KEGG pathways. The significant KEGG pathways of up-regulated DEGs (S3 Fig) within the alkali-stress treatment under 48 h were mostly enriched in plant hormone signal transduction, phenylpropanoid biosynthesis, protein processes, plant-pathogen interaction, and MAPK signaling; the significant KEGG pathways of down-regulated DEGs (S4 Fig) were more in motor protein, photosynthesis, ribosome, carbon metabolism, and cofactors biosynthesis.

### Transcription analysis of hub DEGs

**Fig 7:**
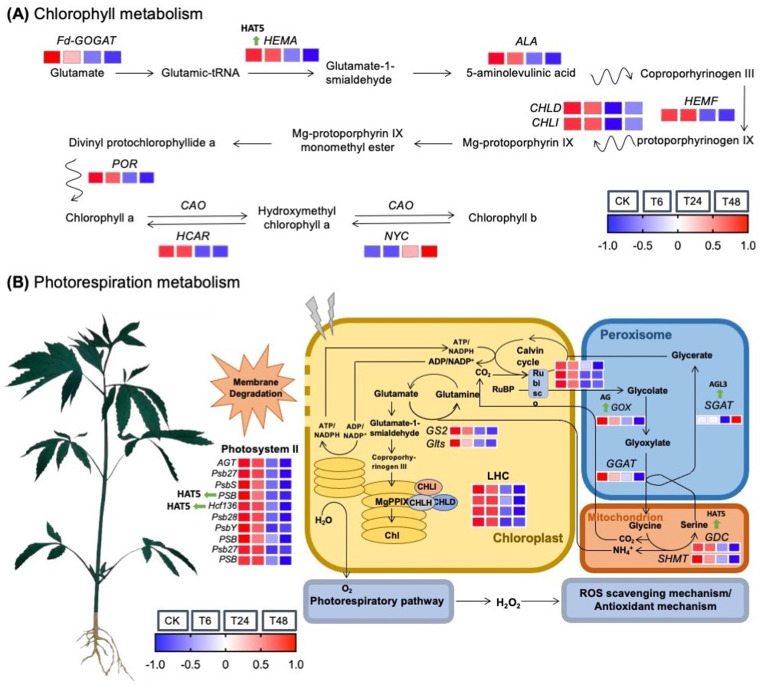
A schematic overview of genetic regulatory mechanisms against effect of alkali-stress on hemp seedling leaves. (A) Chlorophyll metabolism, (B) Photorespiration metabolism. The expression profiles of putative DEGs contributed in the pathway are shown by heat map, respectively.

We extracted a total of 33 hub genes associated with photosynthesis (S2 Table) and also analyzed the photoinhibition effect of alkali-stress on hemp seedling leaves. However, a total of 9 major genes related to chlorophyll metabolism were identified (S3 Table), and their associated transcripts were exhibited by visualized heat map with gradient colors (Fig 7), respectively.

**Fig 8:**
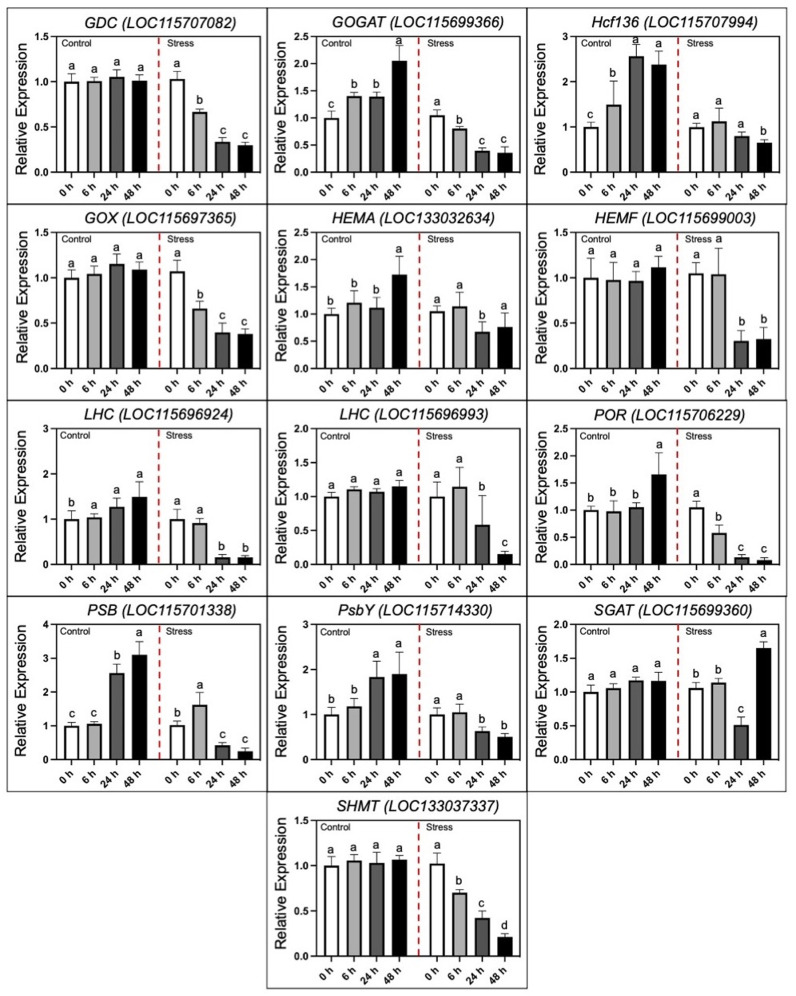
qRT-PCR analysis of identified hub DEGs. The expression verification of 13 DEGs of photosynthesis linked chlorophyll synthesis and photorespiratory metabolism. Statistical letters exhibit the significant differences among expression levels in comparative groups of control (CK) and alkali-stress (NaHCO_3_ (250 mmol·L^−1^)) treatments at different time intervals, respectively.

Then, qRT-PCR analysis revealed expression verification of hub DEGs under control and alkali stress (Fig 8). The expression level of *Fd-GOGAT* gene (*LOC115699366*), which encodes ferredoxin-dependent glutamate synthase 1, and *HEMA* gene (*LOC133032634*), which encodes glutamyl-tRNA reductase (GluTR), catalyzes the biogenesis of glutamate and tetrapyrrole in plastids, triggering the accumulation of chlorophyll contents, which was reduced considerably with the extreme alkali-stress, indicating that substrate accessibility for biosynthesis of chlorophyll is limited. Likewise, stress conditions sup-pressed the expression trends of photosynthesis-like DEGs (*LOC115696924* and *LOC115696993*) in the LHC families, whose products are certain to Chl a and b and transfer excitation energy to the PSI and PSII reaction centers, which was also consistent with the physiological alterations.

Additionally, the mRNA frequency of the 4 genes in the LHC family and 10 genes in photosystem II (PS II) showed a widely down-regulated trend, which may indicate that the stabilization of PS II has been destroyed significantly by alkali-stress. Mean-while, we detected total 10 genes linked with photorespiration. The expression trends of the photorespiration-related four genes (*GOX* (*LOC115697365*), *GDC* (*LOC115707082*), *GGAT* (*LOC115699366*), and *SHMT* (*LOC133037337*)) showed a declining trend under stressed conditions, which was similarly aligned with the above-mentioned genes. However, we noticed that the decreasing rate of these photorespiration-related genes (*GOX* (*LOC115697365*) and *GDC* (*LOC115707082*)) showed an obviously slower trend at 24 ~ 48 h of stress when compared to the initial stress stage of 0 ~ 6 h, and the transcript tendency of the *SGAT* gene (*LOC115699360*) even upsurged in higher ratio relative to control treatment. Thus, we suspected that the inhibitory effect of alkali-stress may have activated the repaired and salvage pathway of photorespiration. The motif analysis and transcription factor information of the predicted hub genes in hemp under alkali stress can be seen in S4 Table and S5 Fig.

## Discussion

Hemp is known as an industrial crop but alkaline stress greatly effects the significant activity of photosynthesis and inhibits essential vegetative growth, reduces plant biomass, and lowers biological yield [3]. There is a dire need to understand the functional genes and pathways associated with alkali stress tolerance that regulate proper plant growth activities. Herein, based on the establishment of a reference genome of industrial hemp, transcriptomic sequencing data were used to evaluate the metabolic pathway and genes associated with regulation of photosynthesis and physiological-related indices in the hemp plant under alkali stress.

The thylakoid membrane is a system of interconnected membranes in chloroplasts that carry out light-dependent photosynthetic reactions necessary for pigmentation and protein complexes to capture sunlight energy and convert it into chemical energy, producing ATP and NADPH [5]. Herein, we observed an internal ultrastructure of sampled hemp leaves and it was noticed that the quantity of granular lamellae decreased in the affected chloroplast under alkali stress as compared to the control (Fig 2), which is consistent with the endogenous fluctuations in the physiological parameters (Figs 3–5) and with the expression patterns of the PSB and PsbY-linked genes involved in light-dependent reactions in PSI and PSII systems (Figs 7–8). However, the inhibitory mechanism of alkali-stress on the thylakoid is not restricted to its structure but also includes the PSII reaction center complex. The PSII reaction center may be the major site of injury from a variety of stress circumstances. The earlier research found that thylakoid membranes in melon can become severely super-oxidized due to salt-induced stress [5], considerably reducing the ability of light capturing and converting into usable chemical energy. Furthermore, it was found that PSII system in pepper plants is consistently pho-to-inhibited during cold stress [51]. However, chlorophyll b is an important element of the light-harvesting complex (LHC), and changes in LHC, usually linked to changes in Chl b level, lead to a significant decline in the enriched thylakoid grana [52]. As a result, we hypothesized that the down-regulated LHC genes (*LOC115696924* and *LOC115696993*) (Figs 7–8), together with the lower chlorophyll b content, have a comprehensive effect on thylakoid formation.

The membrane system acts as an important barrier protecting the cell from exterior stimuli owing to its distinct configuration, vital permeability, and intrinsic lipidic components [53]. Plant-membrane-associated lipids are ordered into 3 different types due to particular processes and architecture. Glycerolipids are basic lipids comprised of distinct fatty acids in glycerol structure [54–57]. Digalactosyldiacylglycerol (DGDG) and monogalactosediacylglycerol (MGDG) primarily form the leaves tissues and assist to modulate the biosynthesis of chloroplast structure and chlorophyll pigment [6]. However, significant remodeling in membrane lipids will occur due to various environmental fluctuations [58]. Herein, the MGDG and DGDG contents were decreased (Table 1), indicative of severe damage that the photosynthetic membranes suffered, and It was discovered that lowering glycolipid levels also reduced chlorophyll content in the leaves [59]. Meanwhile, previous research demonstrated that the ratio of MGDG to DGDG effectively stabilizes the permeability of photosynthetic membranes [5,60], and changes in membrane lipid composition are a possible effort to respond injury to the activity of photosynthesis membrane under phase of stress [61]. For example, a lower ratio of MGDG to DGDG contents during cold-induced stress improves membrane strength for biological functions [7,8]. In contrast, the observed elevated ratio of MGDG to DGDG contents in our results (Table 1) revealed that alkali-stress had a deleterious impact on plastid membrane composition, resulting in uneven photosynthetic homeostasis.

Chlorophyll is a green color pigment that provides plants their green hue and aids in the production of organics via photosynthesis and the development of internal pigment protein complexes. Herein, the chlorophyll content of hemp reduced significantly, as did transpiration, net photosynthesis (CO_2_) concentration, and stomatal shrinking (Fig 4), which was consistent with the alteration observed in numerous plants under stress conditions [62,63]. The biosynthesis process of chlorophyll is dependent on the participation of several genes and proteins, and genetic differences can have an adverse impact on biosynthesis of chlorophyll. The recent results showed that the bio-synthesis of tetrapyrrole pathway is a prominent action through Chl accumulation [64]; however, magnesium chelase (MgCh) and 5-aminolevulinic acid (ALA) are critical sites in regulating the process of chlorophyll modulation. MgCh is complex component made up of CHLI, CHLP, and CHLD that is primarily involved in the primary phase of the branch reaction during biosynthesis of chlorophyll [65]. The *HEMA* gene also encodes GluTR, which initiates the first enzymatic phase of tetrapyrrole biosynthesis in the cytoplasm and chlorophyll formation takes place [66]. In this study, we noticed that the considerable decrease in expression trend of *HEMA* gene (*LOC133032634*) may have resulted in steady biosynthesis of chlorophyll and lower chlorophyll content during alkali stress. In terms of genetic regulation, we discovered that the putative genes (*GOGAT*, *HEMA*/*F*, *ALA*, *CHLI*/*D*, and *POR*) with lower transcript levels influence chlorophyll synthesis degradation in a comprehensive manner (Fig 7). It has been stated that the reduction of photosynthetic genes in response to alkali stress is a type of regulatory mechanism. E.g., Chaves et al. [67] reported that salt stress can trigger rapid gene expression responsible for reprogramming in plants. Even under mild stress, it can optimize resource allocation by down-regulating photosynthetic genes (such as those related to light reaction and Calvin cycle), and this process has no direct association with cell death. The research found through transcriptome analysis that the down-regulation of photosynthetic genes under salt stress is the result of systemic regulation rather than passive cell damage. Yang et al. [68] took sweet sorghum as a model, and proved that the down-regulation of photosynthetic gene expression under salt stress is an adaptive strategy, maintaining cellular carbon balance by protecting the structure of the light system and enhancing the ability of sucrose synthesis. In the current research, the down-regulated photosynthetic genes, e.g., *HEMA*, *GOGAT*, and *POR*, were consistent with the previous studies revealed that the down-regulation of photosynthetic genes is an active regulatory strategy of plants in response to the stressful conditions, rather than the result of passive damage. Moreover, we checked the in silico validation, through promoter motif analysis and transcription factor information, of the predicted hub genes in hemp under alkali stress. The obtained results (see S5 Fig and S4 Table) suggested that most of the hub genes were combined with the HAT5 from the HD-Zip TF family, which has been widely reported to play an important role in enhancing the resistance adversity in many plants, as previously reported. Liu et al. [69] reported that HAT5 enhances the antioxidant capacity of *Arabidopsis thaliana* under drought and salt stress by directly binding to the promoters of superoxide dismutase (SOD) and catalase (CAT) genes. Pan et al. [70] stated that the overexpression of tomato SlHAT5 reduces the activity of antioxidant enzymes and chlorophyll content under high-temperature stress, which may contribute to the inhibition of SuMOylation modification of HSFA1. Thus, it highlights the importance of our research to study the key transcription factors (TF) in hemp under alkali stress for further understanding its resistance mechanism.

The chloroplast and chlorophyll degradation is accelerated during senescence or stress conditions, whereas higher carotenoids level protect the photosynthetic tissues from significant injury, maintain chloroplast integrity, and promote antioxidant activity [71–73]. However, the chloroplast is considered as a key source of ROS production, which promote the accumulation of MDA in plant cells [74,75]. Herein, alkali-induced stress caused significant increase in MDA content (Fig 5) that directly damaged the membrane system of photosynthetic organelles. In addition, we found that carotenoids synthesis was maximized at the start of stress phase as compared to constant reduction in chlorophyll contents (Fig 3), that is related to the previous specified result [50]. However, the protective property of carotenoid showed significant decline and reached its minimum limit due to the steady increase in time interval of alkali-stress (Fig 3).

Alkali-saline treatment induced osmotic stress directly effects the transpiration rate, causes stomatal closure, decreases ambient CO_2_ supply for Calvin cycle, and comprehensively result in severe disturbance in photosynthesis metabolism [76–79]. Herein, our cytological observation of degradation in starch granule (Fig 2) and chlorophyll (Chlf) results (Figs 2–3) indicated that the reduction in stomatal conductance under such water deficit states reduces intercellular CO_2_ levels within leaves, and facilitate the increase in photorespiration. Abiotic stress, such as high irradiance upsurged temperature and closed stomata inhibits CO_2_ absorption for the Calvin cycle. However, the photo respiratory regulation mechanisms for releasing the photoinhibition effect that restrict the availability of CO_2_ to photosynthesis could be distinguished into three aspects. First, it contributes to energy dissipation by directly utilizing the existed reducing power and regenerating the energy acceptors (ADP, NADP+ and NAD+) [21]. Second, it produces the amino acid glycine that can be used for synthesis of glutathione which serves as a carbon donor in the glycine decarboxylase (GDC) reaction, emitting one molecule each of CO_2_ and NH_3_ and as a carbon acceptor in the serine hydroxy methyltransferase (SHMT) reaction, producing serine [80]. Third, it can release CO_2_ restriction in the Calvin cycle due to its intracellular re-cycling within the cells by enhancing the activity of Rubisco [81].

In crop plants, transgenic plants of Arabidopsis depicted photorespiratory enzymes and confirmed maximum efficacy of photosynthesis related growth activities (GDC-H and GDC-L) [82,83], SHMT1 in rice [84], and SGAT in duckweed [85]. Furthermore, previous research found that the proportion of photosynthetic electrons dissipated by reduced CO_2_ adaptation while photorespiration upsurged in tomato production under water stress [86], implying that photorespiration can play significant and positive role in energy dissipation pathway for protecting the photosynthetic apparatus from photoinhibition during drought stress. In grapes, chlf results showed that photorespiration boosted under mild water stress, contributing to the comparatively high photochemical efficiency of the PSII system and the prompt recovery of net photosynthesis rate after irrigation [87]. Herein, the decreased glycine and serine content (Table 2) along with the downregulated transcript and expression level of the GOX, GDC, SHMT and Rubisco activities associated genes under severe alkali stress, signifies that the photorespiratory pathway is regulated by tight signaling cascade that negatively responds to stress conditions. However, the up-regulated *SGAT* gene (*LOC115699360*) probably in an attempt to activate the antioxidant system.

The increasing accumulation of ROS is a typical feature when plants suffered from stress conditions. Furthermore, photorespiration is a vital metabolic activity that triggers the ROS synthesis through the photosynthetic electron transport (PET) chain, with a large amount of hydrogen peroxide (H_2_O_2_) are formed in peroxisomes [88]. Herein, the increased MDA and H_2_O_2_ content demonstrated that alkali-stress stimulated severe oxidative damage to hemp leaves (Fig 5). However, the dual character of photorespiration makes it worthy role in maintaining ideal redox status through ROS scavenging and signaling systems. Since photorespiration can accumulate more >70% of total H_2_O_2_ generated under drought stress, photo respiratory H_2_O_2_ can be a major signal of stress for the induction of scavenging mechanisms resulting in ROS detoxification as well as regulates the redox states of antioxidant activities in leaves [89,90]. In addition, photorespiration not only acts as an indication for the adaptation to drought, but also provides glycine for glutathione synthesis and serine:glyoxylate aminotransferase (SGAT) which catalyze peroxisomal photo respiratory enzymes to provide protection against stress [25].

Herein, our transcriptomic analysis categorized different DEGs related to hemp leaves under comparative groups of control and stress treatments (Fig 6A–6E), and identified DEGs in T48 group of alkali stress (Fig 6F) exhibited a significant alteration trend at the transcriptomic level under alkali stress, that were enriched in, e.g., Galactose metabolism, Glycerolipid metabolism, and Starch and sucrose metabolism which are closely related to the membrane component and starch and sucrose metabolism. Meanwhile, the up-regulated transcript level of the genes enriched in the pathways (e.g., *LOC115697215* (GDP-L-galactose phosphorylase) and *LOC115699196* (*α*-amylase) catalyzing the degradation of galactose and starch) confirmed the physiological observations. Further, the glutathione, POD activity and SGAT activity along with the glycine and serine content seemed to be increased to different extend at initial stage under alkali-stress (Table 2), indicated that the photorespiratory ROS activate the antioxidant system, while the above-mentioned physiological indicators were inhibited by the severe alkali-stress level. Further, in our study, the function of the selected hub genes (Fig 7) regulating the pathways were verified through qRT-PCR (Fig 8). These identified genes have been primarily verified in some of the reference plants under stress phase. For example, Timm, et al. [91] found that through overexpression of the phosphoglycolate phosphatase (PGP) gene that accelerating the degradation of 2-phosphoglycolate (2-PG) could enhance the tolerance of *Arabidopsis thaliana* to high light, high temperature and drought. The transgenic lines maintained higher photosynthetic efficiency and starch accumulation under stress, indicating that the increase in photorespiration flux is a key protective mechanism. Ma, et al. [92] revealed that the photorespiration efficiency may enhance under high pH and improve the expression level of SGAT to mitigate the oxidative stress in millet. Meng et al. [9] reported that the transcriptomic level of *HEMA*, *POR* and *CHLH* declined significantly in wheat under alkali stress. In our experimental study in hemp, the transcript data and qPCR expression (Fig 8) revealed the accuracy of up-regulated expression of candidate *SGAT* gene (*LOC115699360*) associated with the changes in photorespiratory enzymes, which may comprehensively suggested the prominence of photorespiratory pathway in defending plants from stress induced crucial damage. Thus, we suggest that our possibly analyzed genes related to photosynthesis and photorespiration can be utilized for improving the resistance of hemp plants under alkali stress.

## Conclusion

Overall, we observed that morphological and cytological alterations induced by alkali stress indicated the dysfunction of photosynthetic organelles structure, degraded endogenous pigmentation, low chlorophyll contents, and excessive ROS generated by the super-oxidized membrane induced oxidative stress, thus inhibiting the metabolism process of photosynthesis. Transcriptomic sequencing and qPCR verification revealed that the predicted hub genes coding the substrate for chlorophyll synthesis and photosystem were down-regulated, indicating that severe photoinhibition occurred due to alkali stress. However, the up-regulated *SGAT* (*LOC115699360*) gene may have promoted the protective function of photorespiration in stabilizing cellular carbon balance and antioxidant mechanism. Thus, we supposed that identified genes can play a pivotal role for the photorespiration-related genome editing research, which could be an effective orientation to cultivate alkali-tolerant plants of industrial hemp.

## Supporting information

S1 Fig GO enrichment of up-regulated DEGs. (A) Up-regulated genes under alkali stress at 6 h. (B) Up-regulated genes under alkali-stress at 24 h. (C) Up-regulated genes under alkali stress at 48 h.
(DOCX)

S2 Fig GO enrichment of down-regulated DEGs. (A) Down-regulated genes under alkali-stress at 6 h. (B) Down-regulated genes under alkali-stress at 24 h. (C) Down-regulated genes under alkali-stress at 48 h.
(DOCX)

S3 Fig Top 20 KEGG pathways enriched in the upregulated DEGs. (A) Up-regulated DEGs under alkali-stress at 6 h. (B) Up-regulated DEGs under alkali-stress at 24 h. (C) Up-regulated DEGs under alkali-stress at 48 h.
(DOCX)

S4 Fig Top 20 KEGG pathways enriched in the down-regulated DEGs. (A) Down-regulated DEGs under alkali-stress at 6 h. (B) Down-regulated DEGs under alkali-stress at 24 h. (C) Down-regulated DEGs under alkali-stress at 48 h.
(DOCX)

S5 Fig The detailed motif information of the 6 hub genes. (A) Predicted transcription factor family and TF binding sites. (B) Motif analysis exhibited by MEME suite.
(DOCX)

S1 Table The quality and mapping of transcriptomic data. Statistic of sequencing reads obtained from samples of comparative treatments of CK and alkali-stressed groups.
(DOCX)

S2 Table The identified hub DEGs linked to activity of photosynthetic contents. DEGs related to chlorophyll metabolism, photorespiration, and photosynthesis.
(DOCX)

S3 Table The information of exported primers of candidate DEGs. Primers related to chlorophyll synthesis, photo respiratory metabolism, and photosynthesis.
(DOCX)

S4 Table The motif analysis and transcription factor (TF). Information of motif and TF information of the hub genes in hemp under alkali stress.
(DOCX)
