# Supplementary material for: Transcriptomic sequencing and expression verification of identified genes modulating the alkali stress tolerance and endogenous photosynthetic activities of industrial hemp plant
Source: PLoS One. 2025 Jun 25;20(6):e0326434. doi: 10.1371/journal.pone.0326434 (PMC12194151; doi:10.1371/journal.pone.0326434)
Supplement: S2 Fig — (A) Down-regulated genes under alkali-stress at 6 h. (B) Down-regulated genes under alkali-stress at 24 h. (C) Down-regulated genes under alkali-stress at 48 h. (DOCX) [file pone.0326434.s002.docx]

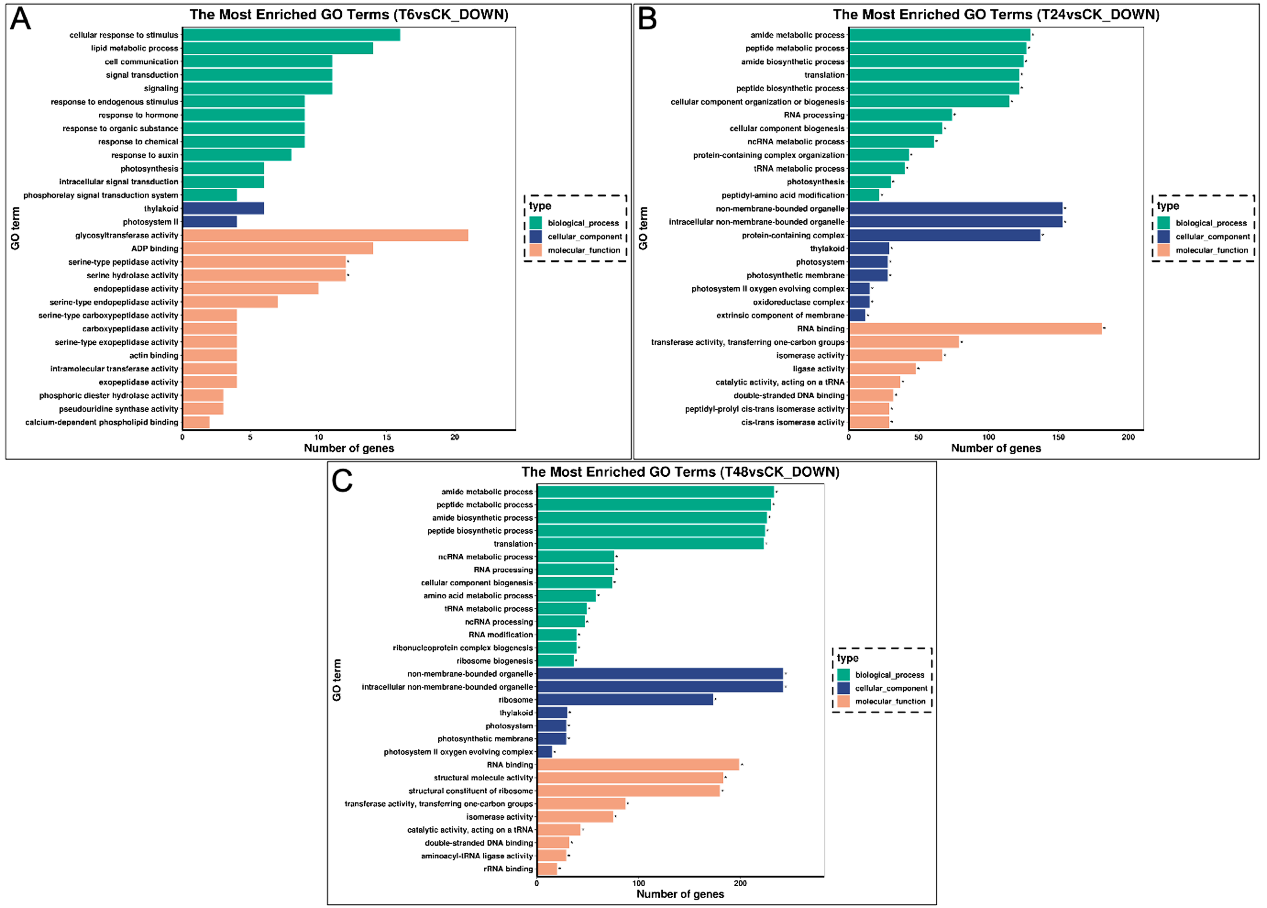


**S2 Fig. GO enrichment of down-regulated DEGs.** (A) Down-regulated genes under alkali-stress at 6 h. (B) Down-regulated genes under alkali-stress at 24 h. (C) Down-regulated genes under alkali-stress at 48 h.
