# Supplementary material for: Transcriptomic sequencing and expression verification of identified genes modulating the alkali stress tolerance and endogenous photosynthetic activities of industrial hemp plant
Source: PLoS One. 2025 Jun 25;20(6):e0326434. doi: 10.1371/journal.pone.0326434 (PMC12194151; doi:10.1371/journal.pone.0326434)
Supplement: S5 Fig — (A) Predicted transcription factor family and TF binding sites. (B) Motif analysis exhibited by MEME suite. (DOCX) [file pone.0326434.s005.docx]

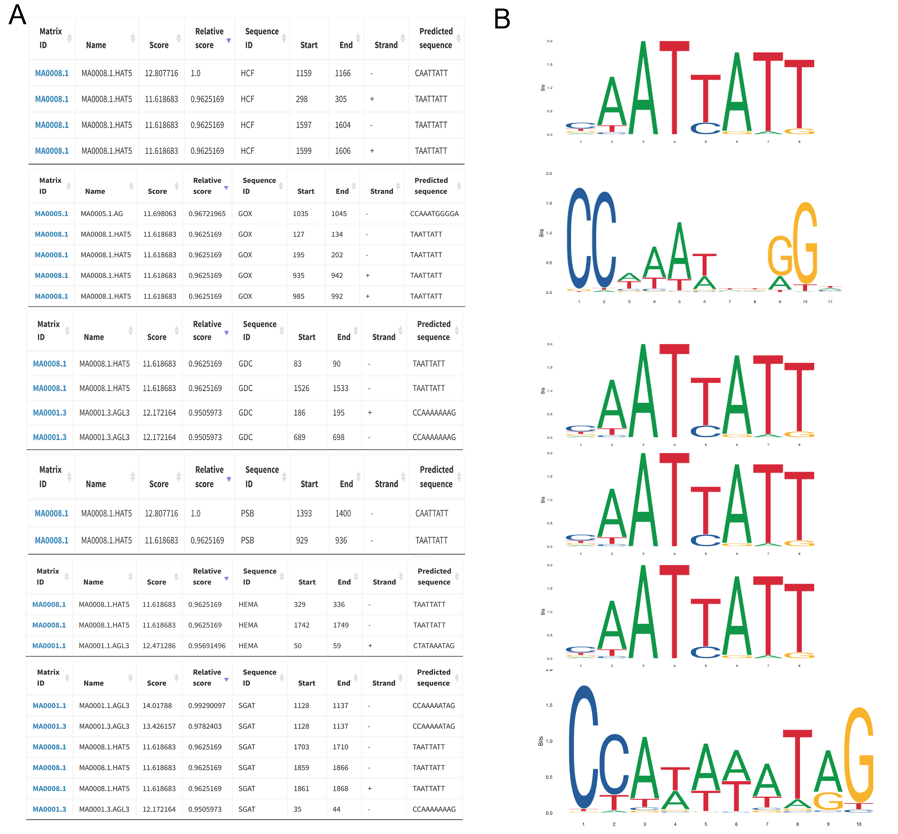


**S5 Fig. The detailed motif information of the 6 hub genes.** (A) Predicted transcription factor family and TF binding sites. (B) Motif analysis exhibited by MEME suite.
