# Supplementary material for: Transcriptomic sequencing and expression verification of identified genes modulating the alkali stress tolerance and endogenous photosynthetic activities of industrial hemp plant
Source: PLoS One. 2025 Jun 25;20(6):e0326434. doi: 10.1371/journal.pone.0326434 (PMC12194151; doi:10.1371/journal.pone.0326434)
Supplement: S1 Table — Statistic of sequencing reads obtained from samples of comparative treatments of CK and alkali-stressed groups. (DOCX) [file pone.0326434.s006.docx]

**S1 Table. The quality and mapping of transcriptomic data.** Statistic of sequencing reads obtained from samples of comparative treatments of CK and alkali-stressed groups.

| **Sample name** | **Raw reads** | **Raw bases** | **Clean reads** | **Clean bases** | **Error rate** | **Q20** | **Q30** | **GC content** | **Total mapped** | **Multiple mapped** | **Uniquely mapped** |
| --- | --- | --- | --- | --- | --- | --- | --- | --- | --- | --- | --- |
| CK_1 | 50606142 | 7.59G | 50125786 | 7.42G | 0.01% | 98.86% | 96.49% | 43.18% | 45765483 (91.3%) | 2240798 (4.47%) | 43524685 (86.83%) |
| CK_2 | 49236674 | 7.39G | 48811792 | 7.23G | 0.01% | 98.89% | 96.60% | 42.68% | 44468677 (91.1%) | 2043163 (4.19%) | 42425514 (86.92%) |
| CK_3 | 55857860 | 8.38G | 55389762 | 8.2G | 0.01% | 98.88% | 96.57% | 42.66% | 50448690 (91.08%) | 2167490 (3.91%) | 48281200 (87.17%) |
| T6_1 | 62363872 | 9.35G | 61779586 | 9.12G | 0.01% | 98.91% | 96.65% | 41.90% | 55803563 (90.33%) | 2462669 (3.99%) | 53340894 (86.34%) |
| T6_2 | 54971004 | 8.25G | 54433190 | 8.03G | 0.01% | 98.89% | 96.63% | 41.55% | 48472924 (89.05%) | 2116790 (3.89%) | 46356134 (85.16%) |
| T6_3 | 52774068 | 7.92G | 52291182 | 7.74G | 0.01% | 98.87% | 96.52% | 43.27% | 47927271 (91.65%) | 2321313 (4.44%) | 45605958 (87.22%) |
| T24_1 | 51952436 | 7.79G | 51312782 | 7.59G | 0.01% | 98.31% | 95.18% | 40.90% | 32105600 (62.57%) | 1283120 (2.5%) | 30822480 (60.07%) |
| T24_2 | 59188732 | 8.88G | 58553928 | 8.66G | 0.01% | 98.53% | 95.77% | 40.14% | 38355696 (65.5%) | 1607164 (2.74%) | 36748532 (62.76%) |
| T24_3 | 57983422 | 8.7G | 57366718 | 8.49G | 0.01% | 98.45% | 95.57% | 39.83% | 36504024 (63.63%) | 1540838 (2.69%) | 34963186 (60.95%) |
| T48_1 | 82528154 | 12.41G | 81164516 | 11.94G | 0.01% | 97.37% | 93.31% | 36.23% | 37585214 (46.31%) | 1887952 (2.33%) | 35697262 (43.98%) |
| T48_2 | 62827674 | 9.42G | 62226116 | 9.23G | 0.01% | 98.89% | 96.61% | 42.21% | 56477828 (90.76%) | 2163777 (3.48%) | 54314051 (87.28%) |
| T48_3 | 65282678 | 9.79G | 64595726 | 9.54G | 0.01% | 98.33% | 95.33% | 40.06% | 44654154 (69.13%) | 1733563 (2.68%) | 42920591 (66.44%) |
