# Supplementary material for: Transcriptomic sequencing and expression verification of identified genes modulating the alkali stress tolerance and endogenous photosynthetic activities of industrial hemp plant
Source: PLoS One. 2025 Jun 25;20(6):e0326434. doi: 10.1371/journal.pone.0326434 (PMC12194151; doi:10.1371/journal.pone.0326434)
Supplement: S2 Table — DEGs related to chlorophyll metabolism, photorespiration, and photosynthesis. (DOCX) [file pone.0326434.s007.docx]

**S2 Table. The identified hub DEGs linked to activity of photosynthetic contents.** DEGs related to chlorophyll metabolism, photorespiration, and photosynthesis.

| **Function** | **Gene Name** | **Gene ID** | **Annotation** |
| --- | --- | --- | --- |
| Chlorophyll metabolism | *GOGAT* | *LOC115699366* | ferredoxin-dependent glutamate synthase 1, chloroplastic/mitochondrial |
|  | *HEMA* | *LOC133032634* | glutamyl-tRNA reductase 1, chloroplastic-like |
|  | *HEMF* | *LOC115699003* | oxygen-dependent coproporphyrinogen-III oxidase, chloroplastic |
|  | *POR* | *LOC115706229* | protochlorophyllide reductase, chloroplastic |
|  | *HCAR* | *LOC115723359* | 7-hydroxymethyl chlorophyll a reductase, chloroplastic |
|  | *ALA* | *LOC115707736* | delta-aminolevulinic acid dehydratase, chloroplastic |
|  | *CHLI* | *LOC115697368* | magnesium-chelatase subunit ChlI, chloroplastic |
|  | *CHLD* | *LOC115718534* | magnesium-chelatase subunit ChlD, chloroplastic |
|  | *NYC* | *LOC115705686* | probable chlorophyll(ide) b reductase NYC1, chloroplastic |
| Photorespiration | *GOX* | *LOC115697365* | glycolate oxidase 1, transcript variant X1 |
|  | *GDC* | *LOC115707082* | glycine dehydrogenase (decarboxylating), mitochondrial |
|  | *SHMT* | *LOC133037337* | serine hydroxymethyltransferase, mitochondrial\|\|serine hydroxymethyltransferase, mitochondrial, transcript variant X2 |
|  | *GGAT* | *LOC115712801* | glutamate--glyoxylate aminotransferase 2, transcript variant X1 |
|  | *Fd-Glts* | *LOC115699366* | ferredoxin-dependent glutamate synthase 1, chloroplastic/mitochondrial |
|  | *GS2* | *LOC115722196* | glutamine synthetase leaf isozyme, chloroplastic |
|  | *SGAT* | *LOC115699360* | serine--glyoxylate aminotransferase |
|  | *Rubisco* | *LOC115695916* | ruBisCO large subunit-binding protein subunit beta, chloroplastic |
|  | *Rubisco* | *LOC115719233* | rubisco accumulation factor 1.1, chloroplastic |
|  | *Rubisco* | *LOC115721093* | ruBisCO large subunit-binding protein subunit alpha |
| Photosystem II | *AGT* | *LOC115696879* | photosystem II reaction center W protein, chloroplastic |
|  | *PSB27* | *LOC115701338* | photosystem II repair protein PSB27-H1, chloroplastic |
|  | *PsbS* | *LOC115702322* | photosystem II 22 kDa protein, chloroplastic |
|  | *PSB* | *LOC115706303* | photosystem II 10 kDa polypeptide, chloroplastic |
|  | *Hcf136* | *LOC115707994* | photosystem II stability/assembly factor HCF136, chloroplastic\|\|photosystem II stability/assembly factor HCF136, chloroplastic, transcript variant X2 |
|  | *Psb28* | *LOC115714005* | photosystem II reaction center PSB28 protein, chloroplastic |
|  | *PsbY* | *LOC115714330* | photosystem II reaction center proteins PsbY, chloroplastic |
|  | *PSB* | *LOC115720918* | photosystem II 5 kDa protein, chloroplastic |
|  | *Psb27* | *LOC115722786* | photosystem II D1 precursor processing protein PSB27-H2, chloroplastic isoform X2\|\|photosystem II D1 precursor processing protein PSB27-H2, chloroplastic, transcript variant X2 |
|  | *PSB* | *LOC133033841* | photosystem II 5 kDa protein, chloroplastic-like |
| LHC | *LHC* | *LOC115696924* | chlorophyll a-b binding protein of LHCII type 1 |
|  | *LHC* | *LOC115696993* | chlorophyll a-b binding protein of LHCII type 1 |
|  | *LHC* | *LOC115699200* | chlorophyll a-b binding protein of LHCII type 1, transcript variant X1 |
|  | *LHC* | *LOC115715576* | protein LHCP TRANSLOCATION DEFECT\|\|protein LHCP TRANSLOCATION DEFECT, transcript variant X1 |
