# Supplementary material for: Transcriptomic sequencing and expression verification of identified genes modulating the alkali stress tolerance and endogenous photosynthetic activities of industrial hemp plant
Source: PLoS One. 2025 Jun 25;20(6):e0326434. doi: 10.1371/journal.pone.0326434 (PMC12194151; doi:10.1371/journal.pone.0326434)
Supplement: S3 Table — Primers related to chlorophyll synthesis, photo respiratory metabolism, and photosynthesis. (DOCX) [file pone.0326434.s008.docx]

**S3 Table. The information of exported primers of candidate DEGs.** Primers related to chlorophyll synthesis, photo respiratory metabolism, and photosynthesis.

| **Function** | **Gene Name** | **Gene ID** | **Annotation** | **Primers sequences (5’-3’)** |
| --- | --- | --- | --- | --- |
| Chlorophyll synthesis | *GOGAT* | *LOC115699366* | ferredoxin-dependent glutamate synthase 1, chloroplastic/mitochondrial | F：GATGCTATGCCACTCAGCCT  R：CGACTGATCCAAGGCAACCT |
|  | *HEMA* | *LOC133032634* | glutamyl-tRNA reductase 1, chloroplastic-like | F：TGTCTCTACAAGTTTCGCCG  R：CCGGCTCATTAGTCTGAACC |
|  | *HEMF* | *LOC115699003* | oxygen-dependent coproporphyrinogen-III oxidase, chloroplastic | F：CTTCATCTTGGCCTATCCGT  R：TATCATCTTCTCGAAGCGCG |
|  | *POR* | *LOC115706229* | protochlorophyllide reductase, chloroplastic | F：TTTGGTTCCCTCTGCTTTCT  R：CTGTGACTATGACACTCCCC |
| Photorespiratory metabolism | *GOX* | *LOC115697365* | glycolate oxidase 1, transcript variant X1 | F：GAGTATGACGCCATTGCAAA  R：TCAGGATGAGCCATCTTCTG |
|  | *GDC* | *LOC115707082* | glycine dehydrogenase (decarboxylating), mitochondrial | F：GGCCAAAAAGTACCGTCAAA  R：ATCTGGTGTTGCCGAGTTAT |
|  | *SHMT* | *LOC133037337* | serine hydroxymethyltransferase, mitochondrial\|\|serine hydroxymethyltransferase, mitochondrial, transcript variant X2 | F：GCTCTCCTCTTCTGTTGACA  R：GTATGAGCTCTAGCCCCTTC |
|  | *SGAT* | *LOC115699360* | serine--glyoxylate aminotransferase | F：TGTAAATGCACCAGGAAGGA  R：TGTAAGTGCACTCTCCCATG |
| Photosystem II | *PSB27* | *LOC115701338* | photosystem II repair protein PSB27-H1, chloroplastic | F：ACTCACACACTCTCTCTCCT  R：GAAAATCAGCGCCGGAAATA |
|  | *HCF136* | *LOC115707994* | photosystem II stability/assembly factor HCF136, chloroplastic\|\|photosystem II stability/assembly factor HCF136, chloroplastic, transcript variant X2 | F：GGCTACTCTCCAACTCACTC  R：GACTTAGCCGGTTGTACAGA |
|  | *PsbY* | *LOC115714330* | photosystem II reaction center proteins PsbY, chloroplastic | F：GCAACAATGGCAATCCTCAA  R：GGTGAATATGGCTCCTGCTA |
| LHC | *LHC* | *LOC115696924* | chlorophyll a-b binding protein of LHCII type 1 | F：GGACGAGTCAACATGAGGAA  R：CTTCAAGCTCACGGTTCTTG |
|  | *LHC* | *LOC115696993* | chlorophyll a-b binding protein of LHCII type 1 | F：TTCCACCATGGCTCTTTCTT  R：AGTTTCTGGGTCAGCTGAAA |
|  | *Actin* |  |  | F：CCAATAGCCTTGCATTCCAT  R：TCGATTGGAAAGCCGAATAC |
