# Supplementary material for: Transcriptomic sequencing and expression verification of identified genes modulating the alkali stress tolerance and endogenous photosynthetic activities of industrial hemp plant
Source: PLoS One. 2025 Jun 25;20(6):e0326434. doi: 10.1371/journal.pone.0326434 (PMC12194151; doi:10.1371/journal.pone.0326434)
Supplement: S4 Table — Information of motif and TF information of the hub genes in hemp under alkali stress. (DOCX) [file pone.0326434.s009.docx]

**S4 Table. The motif analysis and transcription factor (TF).** Information of motif and TF information of the hub genes in hemp under alkali stress.

| Gene Name | Gene ID | Predicted sequence (motif) | TF Family | Transcription Factor |
| --- | --- | --- | --- | --- |
| *HEMA* | *LOC133032634* | TAATTATT | HD-Zip | HAT5 |
| *PSB* | *LOC115701338* | CAATTATT | HD-Zip | HAT5 |
| *HCF* | *LOC115707994* | CAATTATT | HD-Zip | HAT5 |
| *GOX* | *LOC115697365* | CCAAATGGGGA | MADS-box | AG |
| *GDC* | *LOC115707082* | TAATTATT | HD-Zip | HAT5 |
| *SGAT* | *LOC115699360* | CCAAAAATAG | MADS-box | AGL3 |
